# Exploring the therapeutic potential of Saudi *Kleinia anteuphorbium* (L.) DC. Essential oil: Insights into antioxidant, analgesic, and hepatorenal protective effects

**DOI:** 10.1016/j.heliyon.2024.e38573

**Published:** 2024-09-27

**Authors:** Hanan Y. Aati, Mohamed Seif, Mahmoud Emam, Jawaher Al-Qahtani, Hala Attia, Sultan Aati, Juergen Wanner

**Affiliations:** a Pharmacognosy Department, College of Pharmacy, King Saud University. P. O. Box 22452, Riyadh, 11495, Kingdom of Saudi Arabia; b Food Toxicology and Contaminants Department, Food Industries and Nutrition Research Institute, National Research Centre, Dokki, Giza 12622, Egypt; c Phytochemistry and Plant Systematics Department, National Research Centre, Dokki, Giza, 12622, Egypt; d Department of Pharmacology and Toxicology, College of Pharmacy, King Saud University, P.O. Box 2457, Riyadh, 11451, Saudi Arabia; e Dental Health Department, College of Applied Medical Sciences, King Saud University, Riyadh, Saudi Arabia; f Kurt Kitzing Co., Hinterm Alten Schloss 21, D-86757, Wallerstein, Germany

**Keywords:** Analgesic, Carbon tetrachloride, Essential oil, Hepatorenal protection, Hyperpyrexia, *Kleinia anteuphorbium*

## Abstract

Lately, the herb's essential oils have received the intense attention of researchers and pharmaceutical agencies. The chemical composition, antioxidant, analgesic activity, anti-hyperpyrexia, and hepatorenal protective effects of the *Kleinia anteuphorbium* (L.) DC. Essential oil (KAEO) were evaluated. The KAEO was yanked by steam distillation and subjected to a GC/MS system. The antioxidant powers of KAEO were evaluated by DPPH, NO, and FRAP tests. Besides the anti-hyperpyrexia, the analgesic effects were assessed. Moreover, the hepatorenal curative effects were evaluated in a carbon tetrachloride-rat model. Sixty-eight active components were identified by GC/MS. α-pinene was the most dominant monoterpene in KAEO. KAEO showed anti-hyperpyrexia, anti-captive, and great antioxidant powers through DPPH, NO, and FRAP. The oral LD_50_ of KAEO was 187.5 mg/kg in rats. Furthermore, KAEO treatment succeeded in reliving the adverse effects of CCL_4_ in rats as a standard toxic model through reliving the hepatic pathological and oxidative stress and restoring the hepatic and renal functions. Overall, the obtained results demonstrated that KAEO might be used as a potential natural medicine to relieve a variety of modern symptoms and hepatorenal disorders associated with oxidative stress.

## Highlights

• *Kleinia anteuphorbium* essential oil (KAEO) was extracted by hydrodistillation.
• α-pinene was abundant among 68 active metabolites in KAEO *via* GC/MS analysis.
• Oral LD_50_ of KAEO in rats was recorded at 187.5 mg/kg.
• KAEO exhibited high anti-hyperpyrexia, anti-captive, and antioxidant potencies.
• KAEO relieved hepatorenal toxicity in the CCL4-rat model.
• KAEO is a promising natural therapy for oxidative stress-related diseases.

## Graphical abstract

**Figure:**
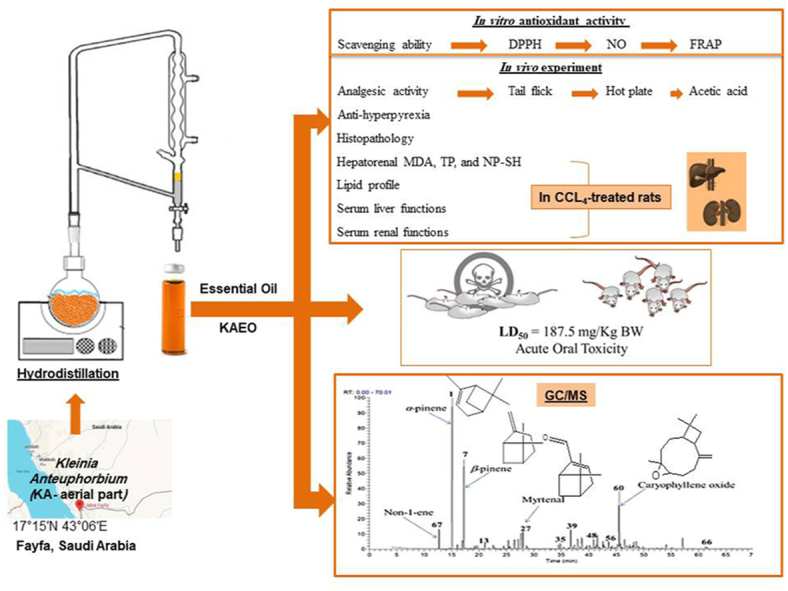

## 1 Introduction

The liver and kidneys are actively involved in metabolic and excretory functions and are common targets of various toxicants. As a vital organ, the liver is mainly responsible for a varied range of reactions essential to human life. The liver is the major site for the metabolism and distribution of nutrients and the detoxification of harmful substances in the body, which may make it susceptible to more suitable for toxic compounds that can an lead to liver injury and eventual dysfunction [1]. Mostly, liver damage is a consequence of exposure to food contaminants and environmental pollutants allied with metabolic function alteration that varies from a transient boost of liver enzymes to hepatic fibrosis, liver cirrhosis, and hepatocellular carcinoma [2]. Additionally, the kidneys play a crucial role in maintaining homeostasis, water and electrolyte balance, detoxification, and excretion of toxic metabolites and drugs. Due to their functions, kidneys are more exposed to harmful substances that can result in renal toxicity [3]. Renal toxicity is characterized by disruptions in electrolyte, urea, and creatinine levels [4].

Nowadays, natural products have attracted the attention of many researchers and become imperative sources of numerous bioactive components that can be used effectively to protect and treat liver and kidney diseases. Many published reports have focused on the therapeutic and protective effects of medicinal herbs and their impact on liver and kidney diseases [[5], [6], [7], [8], [9]]. The Asteraceae family's genus Senecio has a diverse range of secondary metabolism products that are well-known for their several biological properties, including antibacterial [10], antioxidant, and cytotoxic activities [11].

*Kleinia anteuphorbium* (L.) DC. (*K. anteuphorbium*) formerly known as *Senecio anteuphorbuim*, is used in Moroccan folk medicine to cure a variety of illnesses, including burns, rheumatism, stomach, and dorsal pains, as well as wounds and bruises. It is also used as a sedative. Additionally, its juice has been used to soothe skin and eye irritation [12].

Carbon tetrachloride (CCl_4_)-provoked liver injury is an oxidative stress model employed for the assessment of hepatoprotective agents [13]. CCL_4_ is known to cause hepatotoxicity by producing lipid peroxidation and exerting oxidative stress on the organ, which can progress from steatosis to centrilobular necrosis, thereby developing fibrosis and cirrhosis. In the liver microsomes, the trichloromethyl radical (CCl3•), a metabolite (toxic intermediate), is produced when CCL_4_ is detoxified by cytochrome P450, which can induce liver damage [14]. Based on our current understanding, no research about the hepatorenal protection properties of *K. anteuphorbium* L. essential oil (KAEO) has been published so far. The purpose of this experiment was to assess the chemical composition of extracted EO from *K. anteuphorbium* L. in addition to examining its hepatoprotective effects, antioxidant scavenging, analgesic effects, and antipyretic effects.

Carbon tetrachloride (CCl_4_)-provoked liver injury is an oxidative stress model employed for the assessment of hepatoprotective agents [13]. CCL_4_ is known to cause hepatotoxicity by producing lipid peroxidation and exerting oxidative stress on the organ, which can progress from steatosis to centrilobular necrosis, thereby developing fibrosis and cirrhosis. In the liver microsomes, the trichloromethyl radical (CCl3•), a metabolite (toxic intermediate), is produced when CCL_4_ is detoxified by cytochrome P450, which can induce liver damage [14]. Based on our current understanding, no research about the hepatorenal protection properties of *K. anteuphorbium* L. essential oil (KAEO) has been published so far. The purpose of this experiment was to assess the chemical composition of extracted EO from *K. anteuphorbium* L. in addition to examining its hepatoprotective effects, antioxidant scavenging, analgesic effects, and antipyretic effects.

## 2 Materials and methods

### 2.1 Plant materials

In June 2023, aerial sections of Kleinia anteuphorbium (L.) DC. were collected during the floral stage from the Fayfa Mountains in Jazan State (location: 17°15′N 43°06′E). Taxonomist Dr. Rajakrishnan Rajagopal of King Saud University confirmed the botanical identification of the plant. A specimen from the voucher (KSU 24537) has been deposited in the Herbarium at KSU. All protocols involving plants followed the relevant KSU ethical guidelines and regulations (KSU-SE-23-48).

### 2.2 Phytochemical investigation

#### 2.2.1 Extraction and identification of the Kleinia anteuphorbium essential oil (KAEO)

The KAEO was extracted using the hydrodistillation process, as described in prior publications [7,15]. The totaled extracted yield was expressed as a percentage (*v/w*) of dried materials. We passed the resulting KAEOs through Na_2_SO_4_ anhydrous and kept them in an opaque vial at 4 °C until use. Using the gas chromatography/mass spectrometry (GC/MS) approach, as previously defined [16]. The components were identified by comparing their mass spectra to Wiley and NIST library data, standards for the primary components, and Kovat's retention indices to reference libraries [17]. Furthermore, to semi-quantify the components, the normalized peak area of detected components without any adjustment factors were utilized to calculate their relative intensities.

### 2.3 Biological activities

#### 2.3.1 The radical scavenging abilities of KAEO

##### 2.3.1.1 2, 2-diphenyl-2-picrylhydrazyl (DPPH) free radical scavenging assay

The free radical scavenging power of KAEO was calculated agreeing with the method carried out by Alara et al. [18]. The reaction was done between KAEO and a DPPH solute dissolved in methanol, and then the combination was left in the dark for 30 min. Then, the absorbance of the incubated combination was metric at 517 nm by a spectrophotometer (UV–Vis 3000, ORI, Germany). The percentage inhibition was evaluated and calculated using the subsequent equation.

% **inhibition** = 100 × (AC - AS)/AC,

Where AC = Control absorbance and AS = Sample absorbance.

##### 2.3.1.2 Nitric oxide scavenging test

Nitrogen monoxide (NO) released by RAW 264.7 macrophages was considered by quantifying the increase of nitrite, an indicator of NO in the supernatant after 24 h of lipopolysaccharide (LPS) treatment with or without the KAEO using the Griess reagent designated by Mfotie Njoya et al. [19]. Quercetin was introduced as a positive control. The percentage of NO reduction was estimated by comparing the ability of each extract to suppress nitric oxide production by RAW 264.7 macrophages to the control (cells exposed to LPS but not KAEO).

##### 2.3.1.3 Reducing power assay

Depending on the reduction of ferrous ions from Fe (III) to Fe (II), the entire reducing power was detected using the approach proposed by Ref. [20]. The absorbance wavelength was measured at 700 nm against a blank without adding KAEO. Ascorbic acid at varying amounts was used as a standard. The significant absorbance of the chemical process mixture at 700 nm indicates increased reducing power.

### 2.4 Acute toxicity of KAEO

The oral route was used to establish the acute toxicity of the KAEO in accordance with the Organization for Economic Co-operation and Development's recommendations (OECD, 401). Six nominal experimental doses of the tested KAEO (10, 20, 50, 100, 200, and 300 mg/kg BW) were introduced to sixty adult Wistar rats individually over the course of 96 h. The number of breathing, writhing, and sedated piloerection. The 96-h lethal dosage (LD_50_) value was estimated using the logged mortality data using the methodology recommended by Mamza et al. [21].

### 2.5 Anti-hyperpyrexia of KAEO- induced by yeast in rats

Hyperthermia was induced in the deaths and was recorded at 96 h for each treatment. For the first 4 h, the following symptoms were noted: convulsions, tremors, salivation, lethargy, micturition, feces, sleep, and coma in rats by subcutaneous injection of Brewer's yeast (10 mL/kg body weight (BW) and 15 % of aqueous suspension) in the back below the nape of the rat. Before treatment, the rectal temperatures of rats were taken using a thermometer. The control temperatures (pre-drug) were documented 24 h after the yeast injection to define the pyretic reaction of the yeast. 24 h after the yeast injection, the KAEO (5 and 10 mg/kg BW) and the standard medication, paracetamol (150 mg/kg BW), were introduced orally. After the medication therapy, the temperature was measured at 30, 60, and 120 min [22].

### 2.6 Estimation of the analgesic activity of KAEO

#### 2.6.1 Tail flick test

The tail flick test was used to assess the nociception response of KAEO in mice based on the methodology of D'Amour and Smith [23] with slight modifications. Response latencies were measured using a radiant heat automatic tail flick analgesia meter. The tip of the tail (last 1–2 cm) was placed on the heat source, and the endpoint was tail retraction in response to the heat. A 15-s cutoff was used to prevent tail damage from excessive heat. Mice were divided into 4 groups (*n = 6*): normal saline, morphine (10 mg/kg), KAEO at (5 and 10 mg/kg BW). The tail flick latency was recorded at 30, 60, and 120 min after treatment.

#### 2.6.2 Hot plate test

The hot plate assay was utilized to assess the analgesic activity of KAEO in mice, using the described protocol of Eddy and Leimbach [24] with changes. Mice were placed on a hot plate (temperature = 55 ± 1 °C). The time spent paw-licking or hopping was noted. In this test, each mice was separately evaluated on a hot plate to determine the animal's response to electrical heat-induced pain (licking of the forepaws and finally hopping). The period until mice exhibited initial signs of discomfort (hind paw lifting, licking, or jumping) was noted before the starting point. The reaction was measured at 30, 60, and 120 min following the injection of KAEO (5 and 10 mg/kg BW) and morphine (10 mg/kg) as the reference medication.

#### 2.6.3 Acetic acid (CH_3_COOH)-induced writhing response

The writhing test in mice was performed as stated by Koster [25]. Which, male mice (*n = 6*) fasted for 24 h before the experiment and had unlimited access to water. To induce writhing acetic acid (i.p., *v/v*, 0.1 mL/10 g body weight) was administered intraperitoneally. The KAEO (5 and 10 mg/kg BW) was given orally 60 min before the *CH*_*3*_*COOH* injection. Untreated animals exhibiting latencies within 5–30 s were selected. The control group do not treated with KAEO. Indomethacin (10 mg/kg) was given intraperitoneally for 30 min as a positive control before the *CH*_*3*_*COOH* injection. The number of muscle contractions was counted in 5 min after the injection of *CH*_*3*_*COOH* and KAEO at both doses and continued for more than 10 min. The gathered data reflected the total number of writhes seen.

### 2.7 Valuation the hepatorenal-protective activities of KAEO

#### 2.7.1 Animals and experimental design

Forty female Wistar rats, weighing between 175 and 200 g, were obtained from the Experimental Animal Care Center at King Saud University's College of Pharmacy in Riyadh. The animals were housed in polycarbonate cages at the animal facility, where they were fed a regular chow diet in a chemically contamination-free environment with artificial lighting (12 h of light and dark cycles) and temperature maintained at 25 ± 2 °C. The rats were kept in accordance with protocols established by the Decency Committee of the Experimental Animal Care Society at King Saud University's College of Pharmacy in Riyadh, Saudi Arabia (KSU-SF-23-48). CCL_4_ was dissolved in olive oil. After a week of acclimation, the rats were randomly assigned to 5 groups (*n = 8*) for a 4-week treatment regimen [26] as follows.

o **Control group:** untreated group; just feed the normal diet; take laboratory care.
o **CCL**_**4**_**group (CCL**_**4**_**)**: rats treated intraperitoneally with CCL_4_ (1.25 mL/kg BW) twice a week for four weeks.
o **Silymarin/CCL**_**4**_**group (SL/CCL**_**4**_**):** rats administered orally with silymarin (10 mg/kg BW) daily and intraperitoneally with CCL_4_ (1.25 mL/kg BW) twice a week for four weeks.
o **CCL**_**4**_**/*K. anteuphorbium* essential oil** (KAEO-5/CCL_4_): treated orally with essential oil (5 mg/kg BW) daily and intraperitoneally with CCL_4_ (1.25 mL/kg BW) twice a week for four weeks.
o **CCL**_**4**_**/*K. anteuphorbium* essential oil (**KAEO-10/CCL_4_): rats treated orally with essential oil (10 mg/kg BW) daily and intraperitoneally with CCL_4_ (1.25 mL/kg BW) twice a week for 4 weeks.

The blood samples were harvested the day after the final treatment and centrifuged at 3000 rpm for 15 min to remove the serum. Following anesthesia, the animals were euthanized. The liver and kidney tissues were then separated for biochemical and histological analysis.

#### 2.7.2 Hepatic pathological inspection

Liver tissue that had been excised and preserved in formalin was used to make a paraffin block, which was then cut into 4–5 μm slices using a microtome. After fixing the tissue slices on glass slides and staining them with hematoxylin and eosin (H&E), they were inspected *via* an Olympus BX51 light microscope (Tokyo, Japan) with an Olympus E−330 camera [27].

#### 2.7.3 Estimation of serum liver functions marker

Alanine amino transferase (ALT) and aspartate aminotransferase (AST) were assessed based on the methods of [28]. Gammaglutamyl transferase (GGT), alkaline phosphatase (ALP), and bilirubin were calculated as said by Otto et al. [29], Whitfield [30], and Doumas et al. [31] respectively. All supplies were acquired from Roche Diagnostics (GmbH, Mannheim, Germany).

#### 2.7.4 Estimation of serum kidney functions

The serum levels of creatinine, uric acid, and urea were determined according to published methods [[32], [33], [34]], respectively, using Roche kits (Roche Diagnostics GmbH, Mannheim, Germany).

#### 2.7.5 Detection of hepatic and renal MDA, NP-SH, and TP

Liver and kidney tissues were applied to the homogenizing process to prepare tissues homogenate (10 %). The malondialdehyde (MDA) was determined as reported by Ref. [35]. Lowery's approach was used to assess the total protein (TP) level [36]. Furthermore, the non-protein sulfhydryl (NP-SH) was determined using the procedures described by Ref. [37].

#### 2.7.6 Estimation of lipid profile

Total cholesterol, triglycerides, low-density lipoprotein (LDL), and high-density lipoprotein (HDL) levels were detected in serum samples utilizing Roche diagnostic kits (Roche Diagnostics GmbH, Mannheim, Germany) as directed by the manufacturers.

### 2.8 Statistical analysis

The statistics was shown as a mean ± standard error (SE) in the SPSS 11.5 version and statistically analyzed *via* one-way ANOVA and Tukey's test for multiple assessments. Remarkable variances between experimental groups were indicated with a probability value of *P* < 0.05. Figures were designed by GraphPad Prism-9. The *t*-test was used to compare students in Table 4, Table 5, Table 6.

## 3 Results

### 3.1 Phytochemical assessment of extracted KAEO

**Fig. 1:**
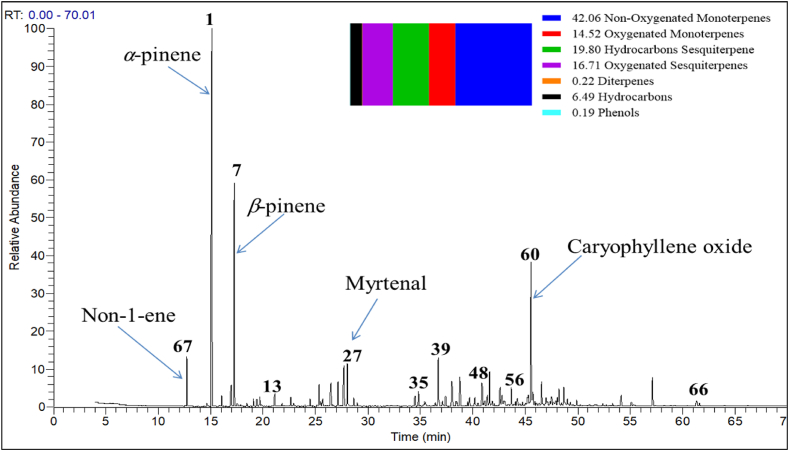
GC-MS chromatogram of KAEO extracted by Hydrodistillation (HD) method. Main components were mentioned. The numbering of peaks depending on table one not depending on the ascending of retention time.

The phytochemical evaluation of KAEO that obtained using hydrodistillation (HD) demonstrated a wide range of volatile compounds, which were estimated based on GC-MS analysis (Fig. 1 and Table 1). The concentration percent of all identified compounds was calculated depending on the percent area of the individual peak toward the total percent peaks of 85.04 % that were detected from the HD technique. GSMS analysis data demonstrated sixty-eight volatile active components, which were characterized as monoterpenes, sesquiterpenes, diterpenes, hydrocarbons, and phenol structures. The concentration of the nonoxygenated monoterpene subclass represented 42.06 % of the total identified compounds. α-pinene structure illustrated the most predominant one in HD EO with 21.24 %. At the same time, oxygenated monoterpene skeletons represented 14.52 %, whereas myrtenal structures illustrated the most dominant one with 3.32 %.

**Table 1:** Percentage chemical composition (peak-area percentage) of KAEO by GC/MS.

NO.	Compounds	t_R_	Conc. %	RI	C.F.
**Non-Oxygenated Monoterpenes (42.06 %)**					
1	α-thujene	14.67	0.14	930	**C**_**10**_**H**_**16**_
2	α-pinene	15.12	21.24	940	**C**_**10**_**H**_**16**_
3	α-fenchene	15.78	0.01	953	**C**_**10**_**H**_**16**_
4	Camphene	15.9	0.08	956	**C**_**10**_**H**_**16**_
5	thuja-2,4(10)-diene	16.09	0.62	960	**C**_**10**_**H**_**14**_
6	Sabinene	16.97	1.21	978	**C**_**10**_**H**_**16**_
7	*β*-pinene	17.27	15.42	984	**C**_**10**_**H**_**16**_
8	myrcene	17.56	0.19	990	**C**_**10**_**H**_**16**_
9	verbenene	18.47	0.19	1009	**C**_**10**_**H**_**14**_
10	δ-3-carene	19.08	0.39	1021	**C**_**10**_**H**_**16**_
11	p-cymene	19.42	0.40	1028	**C**_**10**_**H**_**14**_
12	limonene	19.7	0.76	1034	**C**_**10**_**H**_**16**_
13	Ɣ-terpinene	21.13	0.68	1062	**C**_**10**_**H**_**16**_
14	Terpinolene	22.67	0.73	1093	**C**_**10**_**H**_**16**_
**Oxygenated Monoterpenes (14.52 %)**					
15	1,8-cineole	19.86	0.11	1037	**C**_**10**_**H**_**18**_**O**
16	linalool	22.93	0.24	1098	**C**_**10**_**H**_**18**_**O**
17	perillene	23.09	0.05	1102	**C**_**10**_**H**_**14**_**O**
18	α-fenchol	24.02	0.13	1121	**C**_**10**_**H**_**18**_**O**
19	α-campholenal	24.5	0.44	1131	**C**_**10**_**H**_**16**_**O**
20	nopinone	25.16	0.2	1144	**C**_**9**_**H**_**14**_**O**
21	E-pinocarveol	25.37	1.86	1148	**C**_**10**_**H**_**16**_**O**
22	E-verbenol	25.5	0.31	1151	**C**_**10**_**H**_**16**_**O**
23	p-mentha-1,5-dien-8-ol	26.48	2.05	1171	**C**_**10**_**H**_**16**_**O**
24	borneol	26.66	0.08	1175	**C**_**10**_**H**_**18**_**O**
25	terpinen-4-ol	27.15	1.75	1185	**C**_**10**_**H**_**18**_**O**
26	α-terpineol	27.71	2.8	1196	**C**_**10**_**H**_**18**_**O**
27	Myrtenal	28.07	3.32	1204	**C**_**10**_**H**_**14**_**O**
28	verbenone	28.67	0.66	1216	**C**_**10**_**H**_**14**_**O**
29	carveol	28.98	0.31	1223	**C**_**10**_**H**_**16**_**O**
30	carvone	30.2	0.05	1249	**C**_**10**_**H**_**14**_**O**
31	geranial	30.38	0.02	1253	**C**_**10**_**H**_**18**_**O**
32	phellandral	31.85	0.11	1284	**C**_**10**_**H**_**16**_**O**
33	terpinyl acetate	35.02	0.07	1355	**C**_**12**_**H**_**20**_**O**_**2**_
**Hydrocarbons Sesquiterpenes (19.80 %)**					
34	silphiperfol-5-ene	34.5	0.69	1343	**C**_**15**_**H**_**24**_
35	presilphiperfol-7-ene	34.84	1	1350	**C**_**15**_**H**_**24**_
36	silphiperfol-5-ene	35.38	0.36	1363	**C**_**15**_**H**_**24**_
37	silphin-1-ene	35.53	0.45	1366	**C**_**15**_**H**_**24**_
38	silphiperfola-4,7(14)-diene	35.91	0.06	1374	**C**_**15**_**H**_**22**_
39	silphiperfol-6-ene	36.74	3.72	1393	**C**_**15**_**H**_**24**_
40	modhephene	37.13	0.32	1402	**C**_**15**_**H**_**24**_
41	α-isocomene	37.42	0.76	1409	**C**_**15**_**H**_**24**_
42	α-gurjunene	38.02	2.15	1423	**C**_**15**_**H**_**24**_
43	β-isocomene	38.42	0.27	1433	**C**_**15**_**H**_**24**_
44	cascarilladiene	38.52	0.24	1435	**C**_**15**_**H**_**24**_
45	(E)-β-caryophyllene	38.77	2.25	1441	**C**_**15**_**H**_**24**_
46	isobarbatene	39.7	0.58	1463	**C**_**15**_**H**_**24**_
47	α-humulene	40.22	0.58	1475	**C**_**15**_**H**_**24**_
48	Ɣ-curcumene	40.88	1.89	1491	**C**_**15**_**H**_**24**_
49	α-zingiberene	41.39	0.82	1503	**C**_**15**_**H**_**24**_
50	β-selinene	41.59	2.52	1508	**C**_**15**_**H**_**24**_
51	Ɣ-amorphene	41.87	0.49	1515	**C**_**15**_**H**_**24**_
52	δ-cadinene	42.78	0.66	1538	**C**_**15**_**H**_**24**_
**Oxygenated Sesquiterpenes (16.71 %)**					
53	cameroonan-7-α-ol	42.62	1.16	1534	**C**_**15**_**H**_**26**_**O**
54	silphiperfolan-7-β-ol	42.99	0.15	1543	**C**_**15**_**H**_**26**_**O**
55	presilphiperfolane-9-α-ol	43.08	0.2	1545	**C**_**15**_**H**_**26**_**O**
56	myrtenyl isobutyrate	43.94	0.11	1567	**C**_**14**_**H**_**22**_**O**_**2**_
57	7-epi-silphiperfolan-6-β-ol	44.07	0.16	1570	**C**_**15**_**H**_**26**_**O**
58	AR-turmerol	44.75	0.29	1587	**C**_**15**_**H**_**22**_**O**
59	prenopsan-8-ol	45.22	0.53	1599	**C**_**15**_**H**_**28**_**O**
60	caryophyllene oxide	45.57	10.18	1608	**C**_**15**_**H**_**24**_**O**
61	presilphiperfolan-8-ol	45.74	0.88	1612	**C**_**15**_**H**_**26**_**O**
62	viridiflorol	45.91	0.22	1617	**C**_**15**_**H**_**26**_**O**
63	humulene epoxide II	46.56	1.89	1634	**C**_**15**_**H**_**24**_**O**
64	β-eudesmol	48.1	0.47	1675	**C**_**15**_**H**_**26**_**O**
65	mustakone	49.02	0.45	1699	**C**_**15**_**H**_**22**_**O**
**Diterpenes (0.22 %)**					
66	Manool oxide	61.6	0.22	2049	**C**_**20**_**H**_**34**_**O**
**Hydrocarbons (6.49 %)**					
67	non-1-ene	12.76	6.49	890	**C**_**9**_**H**_**10**_
**Phenols (0.19 %)**					
68	Butylated Hydroxytoluene (BHT)	42.06	0.19	1520	**C**_**15**_**H**_**24**_**O**
	**Total Sum %**		**∼100**		

**t**_**R**_: Retention time, **RI**: Retention Index, **Conc. %**: percent of concentration, **C.**F.: Chemical formula.

In addition, hydrocarbon sesquiterpenes structures represented 19.80 %, whereas silphiperfol-6-ene structures illustrated the most predominant structure with 3.72 %. Oxygenated sesquiterpenes skeletons represented 16.71 %, whereas the caryophyllene oxide structure illustrated the most dominant one with 10.18 %. Likewise, manool oxide, non-1-ene, and butylated hydroxytoluene (BHT) are the only compounds identified as oxygenated diterpene, unsaturated hydrocarbon, and phenol structures, with concentrations of 0.22 %, 6.49 %, and 0.19 %, respectively.

### 3.2 Scavenging activity of KAEO

Data represented in Table 2 indicated that the scavenging activity (%) of extracted EO from KAEO revealed potent scavenging against DPPH, NO, and FRAP with 56.38 ± 7.23, 56.97 ± 6.28, and 0.81 ± 0.06, respectively, in comparison with the scavenging ability of ascorbic acid.

**Table 2:** Scavenging ability of KAEO (%) and IC_50_ (mg/mL).

The scavenging ability of KAEO (%) and IC_50_ (mg/mL)				
		DPPH	Nitric Oxide	Reducing Power (FRAP)
**KAEO**	**10 mg/mL**	12.37 ± 3.68	10.41 ± 2.79	0.43 ± 0.05
	**20 mg/mL**	22.73 ± 4.91	19.31 ± 3.80	0.59 ± 0.03
	**50 mg/mL**	33.65 ± 2.71	47.36 ± 6.22	0.67 ± 0.05
	**100 mg/mL**	56.38 ± 7.23	56.97 ± 6.28	0.81 ± 0.06
	**IC**_**50**_**(mg/mL)**	82.67 ± 15.54	70.54 ± 11.81	–
**Ascorbic acid (100 mg/mL)**		88.57 ± 3.91**∗**	86.69 ± 1.92**∗**	1.76 ± 0.13∗

The data expressed as percent. KAEO = *K. anteuphorbium* essential oil; DPPH = 2, 2-diphenyl-2-picrylhydrazyl; FRAP= Ferric reducing antioxidant power.

### 3.3 Acute oral toxicity of KAEO

Table 3 illustrated the acute toxicity of KAEO. Six groups (each of 10 rats) were administered extracted EO in graduated doses of 10, 20, 50, 100, 200, and 300 mg/kg, respectively. The consequences elucidated that KAEO caused some symptoms such as defecation, sedation, and micturition in the groups administered with 200 and 300 mg/kg BW. The mortality cases were recorded as shown in Table 3 to be used for the valuation of the LD_50_ by Karbar's method. The estimated LD_50_ was 187.5 mg/kg BW.

**Table 3:** The determination of LD_50_ in accordance with *Karbar′s* methodology.

Group	Animals/group (n)	Dose (mg/kg)	Dose differences (a)	Dead animals (n.)	Main of mortality (b)	Probability (a∗b)
I	–	10	–	0	–	–
II	10	20	10	1	0.5	5
III	10	50	30	2	1.5	45
IV	10	100	50	3	2.5	125
V	10	200	100	5	4	400
VI	10	300	150	6	5.5	550
						**1125**

The LD_50_: 300 - (1125/10) = 187.5 mg/kg BW.

**Table 4:** Analgesic activity of KAEO using Tail flick test.

Treatment (Dose)	Pre-drug reaction time (seconds)	Post-drug reaction time (seconds)		
		30 min	60 min	120 min
**Control (normal saline)**	3.93 ± 0.30	4.00 ± 0.31	4.16 ± 0.30	4.13 ± 0.23
**Morphine (10 mg/kg)**	4.00 ± 0.36	5.66 ± 0.33∗∗	6.33 ± 0.33∗∗∗	7.33 ± 0.33∗∗∗
**KAEO (5 mg/kg)**	4.16 ± 0.30	4.33 ± 0.21	4.66 ± 0.33	5.33 ± 0.33∗
KAEO (10 mg/kg)	3.83 ± 0.30	4.16 ± 0.30∗∗∗	5.33 ± 0.33∗∗	6.00 ± 0.13∗∗∗

All values denote the mean ± standard error of the mean (*n = 6*). The statistical significance is indicated by ∗*p < 0.05, ∗∗p < 0.01, ∗∗∗p < 0.001* followed by Student's - *t*-test. The post-drug group was compared with the pre-drug group.

**Table 5:** Analgesic activity of KAEO using Hot plate test.

Group/Dose	Reaction time (seconds, mean ± SEM)			
	Pre-treatment	Post-treatment		
		30 min	60 min	120 min
**Control group**	8.33 ± 0.33	8.50 ± 0.42	8.33 ± 0.49	8.66 ± 0.49
**EO (5 mg/kg)**	8.33 ± 0.42	10.33 ± 0.42∗∗	11.33 ± 0.33∗∗∗	12.50 ± 0.42∗∗∗
**EO (10 mg/kg)**	8.33 ± 0.36	11.66 ± 0.42∗∗∗	12.83 ± 0.47∗∗∗	13.16 ± 0.30∗∗∗
**Morphine (10 mg/kg)**	7.66 ± 0.21	13.00 ± 0.38∗∗∗	14.16 ± 0.30∗∗∗	14.83 ± 0.30∗∗∗

All values denote the mean ± standard error of the mean (*n = 6*). The statistical significance is indicated by ∗*p* < 0.01∗ and ∗∗*p* < 0.001∗∗, with the analysis performed using the Student's t-test. The post-drug group was compared with the pre group.

**Table 6:** Anti-hyperpyrexia of KAEO- induced by brewer's yeast in rats.

Treatment (Dose)	Initial rectal temperature (°C)	Rectal temperature (°C) 5 h after Brewer's yeast induction (°C)	Rectal temperature (°C) after EO administration		
			30 min	60 min	120 min
**Control**	35.08 ± 0.17	38.73 ± 0.17∗∗∗	38.58 ± 0.14	38.55 ± 0.23	38.31 ± 0.1
**KAEO (5 mg/kg)**	35.28 ± 0.18	38.66 ± 0.21∗∗∗	38.46 ± 0.16	38.11 ± 0.10	37.76 ± 0.16∗∗
**KAEO (10 mg/kg)**	35.08 ± 0.18	38.76 ± 0.20∗∗∗	38.10 ± 0.1∗	37.48 ± 0.17∗∗∗	37.20 ± 0.20∗∗∗
**Paracetamol (150 mg/kg)**	35.10 ± 0.15	38.56 ± 0.20∗∗∗	37.58 ± 0.20∗∗∗	36.01 ± 0.12∗∗∗	36.00 ± 0.1∗∗∗

All values represent mean ± SEM (n = 6). *p ∗< 0.05*, *p ∗∗< 0.01*, ∗∗∗*p < 0.001*; followed by student's t-test; pre-drug compared with normal group; post drug compared with yeast administration groups.

### 3.4 Analgesic activity of KAEO

#### 3.4.1 Tail flick examination

The pain threshold was significantly increased after 30 min by the extracted KAEO at doses of 5 and 10 mg/kg. Over various time intervals of observation, KAEO demonstrated a dose-dependent increase in reaction time and showed potent analgesic activity. The results of KAEO's analgesic activity were comparable to the reference standard, as shown in Table 4.

#### 3.4.2 Hot plate experiment

The study uncovered that the treatment of mice with morphine (10 mg/kg i.p.) expanded the inactivity response within the hot plate test. Whereas, the treatment with extracted KAEO at doses of 5 and 10 mg/kg showed a dose-dependent rise within the response time at different time intervals (30, 60, and 120 min) hence treatment (Table 5).

#### 3.4.3 Acetic acid response technique

**Fig. 2:**
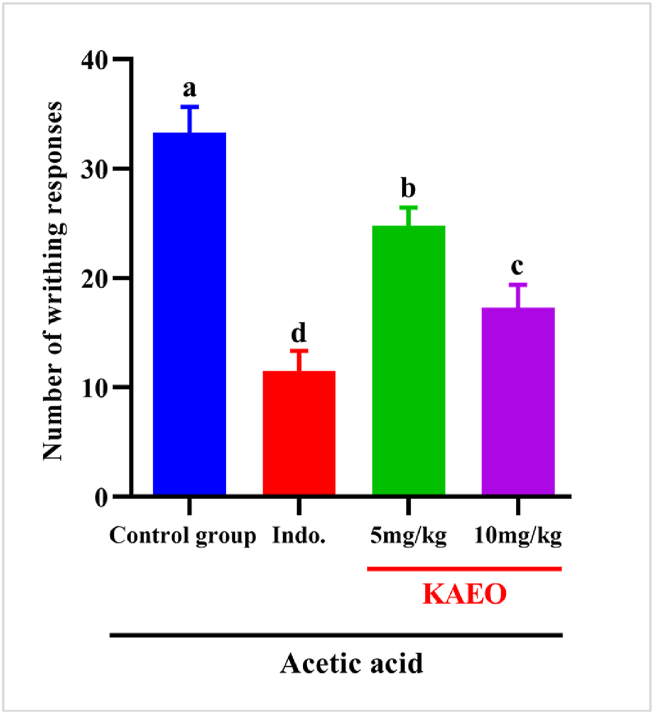
The analgesic effects of KAEO at two doses on mice's acetic acid-induced writhing response. The values are represented as the mean ± SEM (*n = 6*). Distinct letters indicate significant differences between groups at a significance level of *p < 0.05*. Indomethacin (Indo) served as a reference drug in comparison to KAEO.

Fig. 2 unveils that acidic acid-triggered writhing reactions in mice indicate pain-relieving exercises applied by KAEO. The writhing reactions were actuated by intraperitoneal infusion of acidic corrosive within the control bunch. The writhing reactions were seriously decreased by treatment with KAEO at 5 and 10 mg/kg (*p < 0.05*) or indomethacin.

### 3.5 Anti-hyperpyrexia activity of KAEO

The rectal temperature was noticeably increased by a subcutaneous injection of yeast suspension 24 h after delivery. However, the rats' rectal temperatures were dramatically reduced in a dose-dependent manner when they were treated with KAEO at two different dosages (5 and 10 mg/kg BW). Following KAEO treatment, the antipyretic effects lasted for 2 h, starting within the first 30 min. Rats treated with KAEO and conventional treatment (paracetamol) had significantly lower values than the control group, indicating that the yeast-induced elevate in rectal temperature was less severe (Table 6).

### 3.6 Histopathology findings

**Fig. 3:**
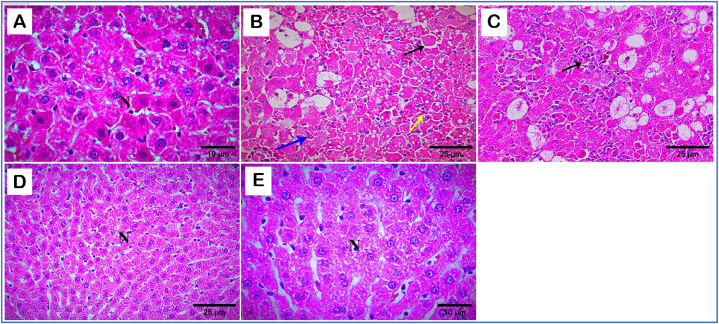
Illustrative (H & E) sections of the liver tissues of rats exposed to CCL_4_ (1.25 mg/kg BW) for 28 days and treated simultaneously with silymarin (10 mg/kg BW) or with KAEO at both doses (5 and 10 mg/kg BW). Control rats. A section of control liver display normal hepatocytes (N) and intact cell membranes [A]. A section of the liver of CCL4-treated rats shows a different hepatocyte structure, with loss of the cell membrane (black arrow), enlarged nuclear size (blue arrow), and infiltration of connective tissue with prominent vacuoles and necrosis (yellow arrow) [B]. In contrast, a slice of the liver of rats treated with CCL_4_ and silymarin shows effective hepatoprotection, with outward arrangement of hepatocytes, intact cell membranes, and relief of necrosis and vacuoles (black arrow) [C]. Additionally, a section of the liver of rats treated with CCL_4_ and low-dose KAEO exhibits a slight hepatoprotective effect, relieving necrosis and vacuolation in hepatocytes (N) [D]. Furthermore, a section of the liver of rats treated with CCL_4_-extracted KAEO at a high dose shows exceptionally hepatoprotective properties, with normal structure of hepatocytes, radially arranged hepatocytes with intact cell membranes, and absence of necrosis and vacuoles (N) [E].

The remedial effects of KAEO against histopathological changes induced by CCL_4_ in rats were assessed through histopathological evaluation. The normal architecture of hepatocytes and clear sinusoids was observed in the hepatic region of the normal control group (Fig. 3A). In contrast, the liver section of CCL_4_-treated rats showed various histological changes, including altered hepatocyte morphology, cell membrane damage, enlarged nuclear size, and connective tissue infiltration with prominent necrosis (Fig. 3B). On the other hand, rats treated simultaneously with CCL_4_ and KAEO at 5 and 10 mg/kg BW showed significant improvement in liver histopathological features compared to the CCL_4_ group (Fig. 3D and E). Rats treated concurrently with Silymarin and CCL_4_ did not exhibit any pathological alterations and showed normal hepatocytes compared to the CCL_4_-treated group (Fig. 3C).

### 3.7 Extracted KAEO restored hepatorenal MDA, TP, and NP-SH in CCL_4_-treated rats

**Fig. 4:**
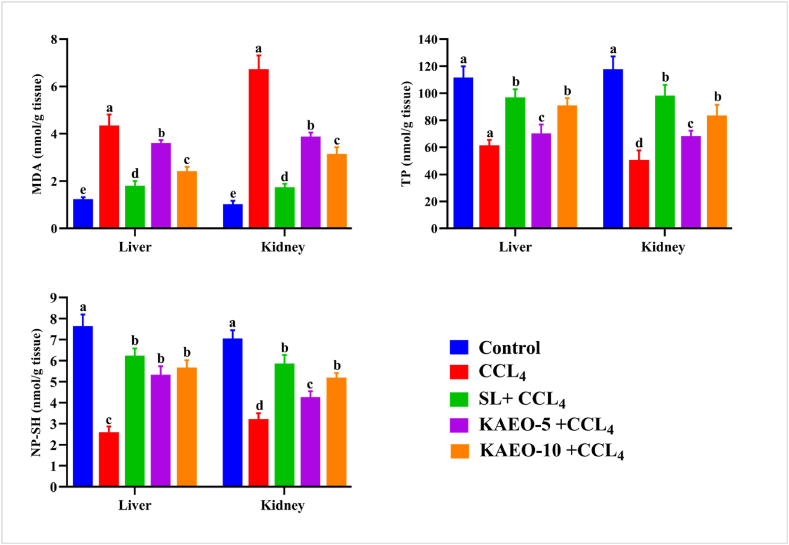
The impact of KAEO at two different doses on liver and kidney levels of MDA, TP, and NP-SH in CCL_4_-treated rats. The presented data is expressed as the mean ± SE (*n = 6*). CCL_4_ = Carbon tetrachloride, SL= Silymarin, KAEO-5 = essential oil at a dose of 5 mg/kg BW, KAEO-10 = essential oil at a dose of 10 mg/kg BW, MDA = Malondialdehyde, TP = Total protein, and NP-SH= Non-protein sulfhydryl. Significant differences between groups are indicated by different letters (*p < 0.05*). SL was utilized as the standard drug.

**Fig. 5:**
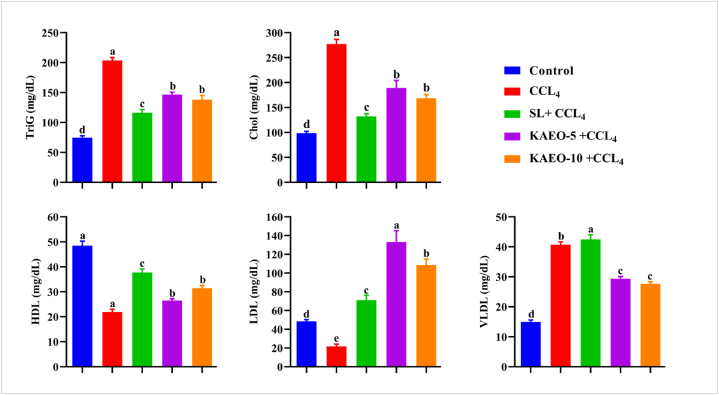
The effects of KAEO on the lipid profile of CCL_4_-treated rats. Data are expressed as mean ± SE (*n = 6*). CCL_4_ = carbon tetrachloride, SL = silymarin, KAEO-5 = essential oil at a dose of 5 mg/kg BW, KAEO-10 = essential oil at a dose of 10 mg/kg BW KAEO, TriG = triglycerides, Chol = cholesterol, HDL = high-density lipoprotein, LDL = low-density lipoprotein, and VLDL = very low-density lipoprotein. Significant differences between groups are indicated by different letters (*p < 0.05*). SL was utilized as the standard drug.

MDA, TP, and NP-SH were measured in liver and kidney tissue homogenates as indicators of oxidative stress. The data presented in Fig. 5 demonstrate that CCL_4_ treatment leads to a significant decrease (*p < 0.05*) in hepatic and renal levels of TP and NP-SH, while also causing notable increases in the lipid peroxidation product (MDA) compared to the control group. Rats treated with KAEO in conjunction with CCL_4_ exhibited remarkable improvements in TP and NP-SH concentrations at both tested doses, as well as substantial recovery in lipid peroxidation, similar to the silymarin-treated group (Fig. 4).

### 3.8 KAEO maintained lipid profile in CCL_4_-treated rats

**Fig. 6:**
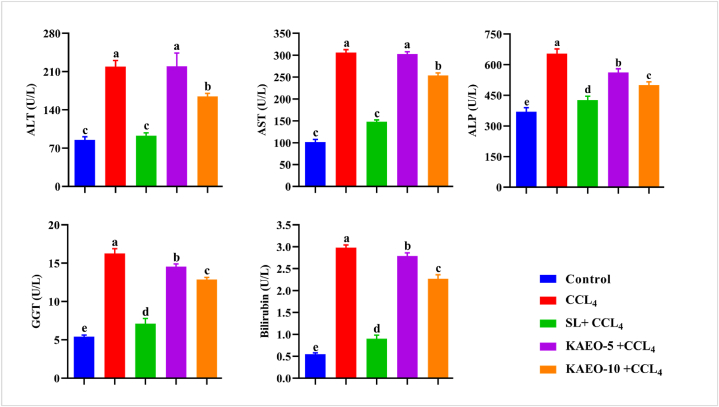
The effect of KAEO on liver function indicators in CCl_4_-treated rats. The data are expressed as mean ± SE (*n = 6)*. CCl_4_ = carbon tetrachloride; SL = silymarin; KAEO-5 = essential oil at a dose of 5 mg/kg BW; KAEO-10 = essential oil at a dose of 10 mg/kg BW KAEO; ALT = alanine transaminase; AST = aspartate transaminase; GGT = gamma-glutamyl transpeptidase; and ALP = alkaline phosphatase. Significant differences between groups are indicated by different letters (*p < 0.05*). SL was utilized as the standard drug.

To assess the palliative effects of KAEO on lipid profile markers in CCL4-treated rats, serum cholesterol, triglycerides (TriG), LDL, HDL, and VLDL were evaluated. The results shown in Fig. 6 demonstrate that CCL_4_ treatment led to a significant increase in total cholesterol, TriG, LDL, and VLDL levels, along with a notable decrease in HDL levels compared to the control group. In contrast, treatment with KAEO and CCL_4_ together resulted in a significant improvement (*p < 0.05*) in the tested lipid profile markers compared to the control group, indicating enhancement of the lipid metabolism in KAEO-treated rats at both doses. While, rats receiving 10 mg of KAEO had significantly lower TriG levels than rats treated with silymarin after exposure to CCL4 (Fig. 5).

### 3.9 KAEO restored serum liver functions in CCL_4_-treated rats

CCL4 therapy caused pathological deviations in the liver, including hepatomegaly and serologic abnormalities, as well as an increase in the activities of ALT, AST, bilirubin, and GGT. Fig. 5 shows that CCL4 injection significantly increased (*P < 0.05*) the activity of ALT, AST, bilirubin, and GGT. Concurrent administration of KAEO at two doses (5 and 10 mg/kg BW) dramatically reduced hepatic function biomarkers to near-normal levels, with the higher dose (10 mg/kg BW) showing greater efficacy than the lower dose (5 mg/kg). A similar preventive effect was observed in rats that received silymarin (Fig. 6).

### 3.10 KAEO relieved serum renal functions in CCL_4_-treated rats

**Fig. 7:**
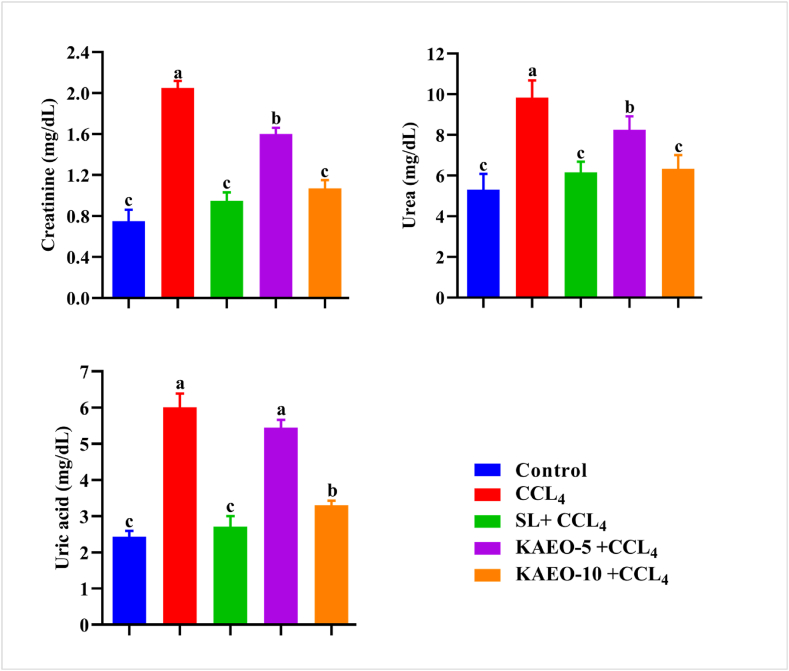
The effect of KAEO on renal function in CCL_4_.- treated rats. The data are expressed as mean ± SE (*n = 6*). CCL_4_ = for carbon tetrachloride, SL = silymarin, KAEO-5 = essential oil at a dose of 5 mg/kg BW, and KAEO-10 = essential oil at a dose of 10 mg/kg BW KAEO. Significant differences between groups are indicated by different letters (*p < 0.05*). SL was utilized as the standard drug.

CCL4-treated rats showed significant increases (*p < 0.05*) in serum urea, uric acid, and creatinine levels. Concurrent administration of KAEO with CCL4 at a high dose (10 mg/kg BW) effectively maintained normal serum urea and creatinine levels, as opposed to the high levels seen in the CCL4 group. However, KAEO at 5 mg/kg BW did not effectively restore blood uric acid compared to the CCL4-treated group (*p > 0.05*, Fig. 7).

## 4 Discussion

The phytochemical constituents of the extracted essential oil from *K. anteuphorbium* were evaluated through GC/MS analysis, as well as the antioxidant, analgesic, anti-inflammatory, and antipyretic activities. Moreover, the hepatorenal protective effects were evaluated using the CCL4 rat model. The results revealed that the extracted EO is rich in essential volatile components, which belong to bioactive classes such as monoterpenes, sesquiterpenes, diterpenes, hydrocarbons, and phenols. These components exhibit various pharmaceutical and therapeutic activities [[38], [39], [40]]. Additionally, the acute toxicity test has shown that KAEO can be safely used up to 187.5 mg/kg BW in rats.

The *K. anteuphorbium* EO has shown effective potential in treating pain, inflammation, and fever in several animal models. Among the spinal antinociceptive reactions, the tail-flick, hot plate, and acetic acid tests were conducted. The hot plate experiment measures animal nociceptive reaction latencies to a thermal stimulus (supraspinal), while the tail-flick test primarily assesses spinal reactions [41,42].

The extracted EO was delayed until the tail immersion event. The tail-immersion action is thought to be a spinally mediated reflex [43]. Harmoniously, the treatment with KAEO extended the reaction time against the hot plate experiment and significantly decreased the writhing number in the rats treated with acetic acid. These outcomes suggest that the peripheral and central mechanisms are included in the antinociceptive activities of the KAEO. Moreover, the analgesic activity of the KAEO was proven, which may be attributed to its active ingredients as such α-pinene, β-pinene, myrtenal, and α-terpineol [44,45]. Hyperpyrexia is a life-threatening emergency; without quick, proper, and urgent treatment, it can cause long-term complications and death [46]. In this regard, the anti-hyperpyrexia potential of the KAEO appeared in rats treated with brewer's yeast. These results provide evidence for the potential of KAEO as an antipyretic for the management of fever due to its antioxidant and anti-inflammatory activity.

CCL_4_ is considered an ideal model for the evaluation of hepatoprotective agents and drugs. Nowadays, natural hepatorenal protectants are becoming highly demanded and promising because they exert safe protective and therapeutic effects without causing dangerous side effects. One of the main sources of natural hepatorenal protectants is the bioactive components that can be extracted from plants and herbs [47]. The maintenance of body homeostasis is one of the prime functions of the liver and kidneys in humans and animals.

In the current study, *K. anteuphorbium* EO (KAEO) exerted defensive effects against the adverse effects of CCL_4_ in rats, as evidenced by a significant reversal of the pathological events induced in CCL_4_-treated rats, particularly in high dose-treated rats (KAEO-10 mg/kg BW). These effects may be attributed to the antioxidants and anti-inflammatory properties generated by its identified bioactive components. These results are consistent with findings from reports evaluating the ability of essential oils in the CCL_4_ model [7].

It's well known that the breakdown in the liver results in oxidative stress overwhelms the antioxidant defense mechanism. The study results revealed that CCL_4_ exposure led to an increase in the generation of lipid peroxide and a decrease in TP and NP-SH levels in the liver and kidney tissues. These findings were consistent with the results of many published reports [[48], [49], [50], [51], [52]]. The administration of CCL_4_ causes degradation of the lipids in the cellular membrane a lipid peroxidation process through its highly active metabolites. This leads to the production of MDA products, which compromise the integrity of the cell membrane and damage the liver [53]. Similarly, MDA is the most common bioindicator used to indicate oxidative stress; it is the end product of lipid peroxidation, which typically triggers the continued and enhanced the propagated and developed damage; impairment; MDA can promote the oxidation of the polyunsaturated phospholipids, leading to impaired membrane function [54].

Similarly, decreasing the TP and NP-SH levels is an ideal indicator of liver injury [55]. A drop in total protein may result in dehydration, which is detrimental to cellular equilibrium and can adversely affect the body's health by impacting the metabolic processes of the liver and kidneys [56]. Improvements in TP levels at two tested dosages of KAEO and silymarin indicate a reduction in oxidative stress, which helps alleviate renal and hepatic damage. KAEO treatment concurrently significantly increased TP and NP-SH levels and decreased lipid peroxidation. The antioxidant activity, supported by studies on the scavenging of free radicals (DPPH, FRAP, and NO), may be responsible for these benefits.

In the present study, the KAEO treatment reinstated the liver function biomarkers (ALT, AST, ALP, GGT, and bilirubin) to the control level, particularly at the high treated dose (10 mg/kg BW) compared to their level in the CCL_4_-treated group. Additionally, the treatment with KAEO during CCL_4_ exposure normalized the levels of lipid profile markers (TriG, Chol, LHD, VLDL, and HDL). Furthermore, the concurrent treatment of KAEO plus CCL_4_ mitigated the adverse effects of CCL_4_ on the kidney's function biomarkers (urea, creatinine, and uric acid) and restored them to positive control limits. The treatment with the high dose (10 mg/kg BW) was had a highly positive influence against the adverse effects of CCL_4_. These current results are good indicators of the hepatoprotective properties of the KAEO.

The tested hepatic and renal function biomarkers are reliable and commonly used for assessing liver and renal injury [57,58]. The elevated serum liver function markers and changes in lipid profile markers were attributed to hepatocyte damage and impaired lipid metabolism [59]. Therefore, the increase in liver damage indicators directly correlates with alterations in the structural integrity of hepatic tissue, as observed histologically. Hepatic damage indicators were significantly reduced, and the liver was successfully protected by the simultaneous administration of KAEO and CCL_4_ at two tested doses.

Among these indicators, the increased release of bilirubin into the bloodstream in CCL_4_-treated animals has been linked to the liver's deteriorating function in maintaining bile [60], while an upsurge in serum values of AST, ALT, GGT, and ALP indicates injuries to the hepatic cells' cell membrane that cause the cells' permeability to be disrupted [61]. Among lipid profiles, high serum cholesterol levels may also be due to hepatic function deficiency [62], whereas the decreased activity of hepatic cytochrome P450 enzymes could also be the reason for increased cholesterol concentration [63]. Triglyceride level is also one of the prime liver harm markers [64]. The alleviation of serum urea, uric acid, and creatinine values is reflected in renal damage biomarkers, which can be induced by increasing the production of lipid peroxidation and decreasing the kidney's total protein [65].

The KAEO was able to restore hepatic and renal function biomarkers back to normal levels. This improvement of liver and kidney functions might be imputed to the treatment of the content of the bioactive components in KAEO, namely the major components, α-pinene, *β*-pinene, carypophellene oxide, silphiperfol-6-ene, myrtenal, α-terpinal, (E)-*β*-caryophyllene, and *α*-gurjunene. Chemically, these components belong to several phytochemical classes like monoterpenes, sesquiterpenes, and diterpenes that were previously reported to exert hepatic and renal protection *via* their antioxidants and anti-inflammatory properties [[66], [67], [68], [69], [70], [71], [72]].

The KAEO was able to restore hepatic and renal function biomarkers back to normal levels. This improvement in liver and kidney functions might be attributed to the treatment of the bioactive components in KAEO, namely the major components: α-pinene, β-pinene, caryophyllene oxide, silphiper-fol-6-ene, myrtenal, α-terpinol, (E)-β-caryophyllene, and α-gurjunene. Chemically, these components belong to several phytochemical classes like monoterpenes, sesquiterpenes, and diterpenes that were previously reported to exert hepatic and renal protection through their antioxidant and anti-inflammatory properties [66–72].

## 5 Conclusion

In summary, the current findings reveal that *K. anteuphorbium* essential oil contains several bioactive components that exhibit significant antioxidant, preventive, anti-hyperpyrexia, and hepatoprotective activities. The LD_50_ of KAEO is approximately 187.5 mg/kg when administered orally in rats, indicating a safe treatment with no changes in behavior. Furthermore, KAEO demonstrated highly hepatorenal protective properties by maintaining body homeostasis, overcoming the elevation of MDA, and restoring liver and kidney functions and lipid profile biomarkers in CCL_4_-treated rats.

## Data availability statement

All data was inserted into our manuscript.

## Ethics statement

All protocols using plants followed relevant KSU ethical guidelines and regulations (KSU-SE-23-48).

## CRediT authorship contribution statement

**Hanan Y. Aati:** Writing – review & editing, Resources, Methodology, Funding acquisition, Formal analysis, Data curation, Conceptualization. **Mohamed Seif:** Writing – review & editing, Writing – original draft, Visualization, Software, Formal analysis, Conceptualization. **Mahmoud Emam:** Writing – review & editing, Writing – original draft, Visualization, Software, Investigation, Formal analysis, Conceptualization. **Jawaher Al-Qahtani:** Writing – review & editing, Supervision, Project administration, Funding acquisition, Formal analysis. **Hala Attia:** Writing – review & editing, Project administration, Methodology, Funding acquisition, Formal analysis. **Sultan Aati:** Writing – review & editing, Resources, Funding acquisition, Formal analysis. **Juergen Wanner:** Writing – review & editing, Software, Resources, Formal analysis, Data curation.

## Declaration of competing interest

The authors declare that they have no known competing financial interests or personal relationships that could have appeared to influence the work reported in this paper.

## References

[1] Lubna S., Ahmad R. (2023). Clinical and biochemical understanding of Zinc interaction during liver diseases: a paradigm shift. J. Trace Elem. Med. Biol..

[2] Bilhartz J., Askari F. (2022). Metabolic diseases of the liver. Yamada's Textbook of Gastroenterology.

[3] Molaei E., Molaei A., Abedi F., Hayes A.W., Karimi G. (2021). Nephroprotective activity of natural products against chemical toxicants: the role of Nrf2/ARE signaling pathway. Food Sci. Nutr..

[4] Obafemi T.O. (2022). Gallic and Hesperidin ameliorate electrolyte imbalances in AlCl 3-induced nephrotoxicity in wistar rats. Biochemistry Research International.

[5] Seif M., Aati H., Amer M., Ragauskas A.J., Seif A., El-Sappah A.H., Aati A., Madboli A.E.-N.A., Emam M.J.M. (2023). Mitigation of hepatotoxicity via boosting antioxidants and reducing oxidative stress and inflammation in carbendazim-treated rats using Adiantum capillus-veneris L. Extract.

[6] Bencheikh N., Elbouzidi A., Kharchoufa L., Ouassou H., Alami Merrouni I., Mechchate H., Es-Safi I., Hano C., Addi M., Bouhrim M. (2021). Inventory of medicinal plants used traditionally to manage kidney diseases in north-eastern Morocco: ethnobotanical fieldwork and pharmacological evidence. Plants.

[7] Aati H.Y., Emam M., Al-Qahtani J., Aati S., Aati A., Wanner J., Seif M.M. (2022). Chemical composition of Tagetes patula flowers essential oil and hepato-therapeutic effect against carbon tetrachloride-induced toxicity (In-Vivo). Molecules.

[8] Seif M., Abd El-Aziz T., Sayed M., Wang Z. (2021). Zingiber officinale ethanolic extract attenuates oxidative stress, steroidogenic gene expression alterations, and testicular histopathology induced by sodium arsenite in male rats. Environ. Sci. Pollut. Control Ser..

[9] Madboli A.E.-N.A., Seif M.M.J.E.S., Research P. (2021).

[10] Ouhaddou S., Aghraz A., Ben Bakrim W., Sissi S., Larhsini M., Markouk M., Bekkouche K., Arrigo S., Cicero N., Costa R. (2022). Analysis of volatiles in Senecio anteuphorbium essential oil with a focus on its allelopathic effect by means of gas chromatography. Separations.

[11] Loizzo M.R., Tundis R., Statti G.A., Menichini F. (2007). Jacaranone: a cytotoxic constituent from Senecio ambiguus subsp. ambiguus (Biv.) DC. against renal adenocarcinoma ACHN and prostate carcinoma LNCaP cells. Arch Pharm. Res. (Seoul).

[12] Elhidar N., Nafis A., Kasrati A., Goehler A., Bohnert J.A., Abbad A., Hassani L., Mezrioui N.-E. (2019). Chemical composition, antimicrobial activities and synergistic effects of essential oil from Senecio anteuphorbium, a Moroccan endemic plant. Ind. Crop. Prod..

[13] Wang M., Niu J., Ou L., Deng B., Wang Y., Li S. (2019). Zerumbone protects against carbon tetrachloride (CCl4)-induced acute liver injury in mice via inhibiting oxidative stress and the inflammatory response: involving the TLR4/NF-κB/COX-2 pathway. Molecules.

[14] Iqbal N., Zubair H.M., Almutairi M.H., Abbas M., Akhtar M.F., Aleya L., Kamel M., Saleem A., Jabeen Q., Noreen S. (2022). Hepatoprotective effect of Cordia rothii extract against CCl4-induced oxidative stress via Nrf2–NFκB pathways. Biomed. Pharmacother..

[15] Aati H.Y., Attia H., Babtin R., Al-Qahtani N., Wanner J. (2023). Headspace solid phase micro-extraction of volatile constituents produced from Saudi ruta chalepensis and molecular docking study of potential antioxidant activity. Molecules.

[16] Aati H.Y., Perveen S., Orfali R., Al-Taweel A.M., Aati S., Wanner J., Khan A., Mehmood R. (2020). Chemical composition and antimicrobial activity of the essential oils of Artemisia absinthium, Artemisia scoparia, and Artemisia sieberi grown in Saudi Arabia. Arab. J. Chem..

[17] Adam M., Phillips M., Blok V. (2007). Molecular diagnostic key for identification of single juveniles of seven common and economically important species of root‐knot nematode (Meloidogyne spp.). Plant Pathol..

[18] Alara O., Abdurahman N., Mudalip S.A., Olalere O. (2019). Effect of drying methods on the free radicals scavenging activity of Vernonia amygdalina growing in Malaysia. J. King Saud Univ. Sci..

[19] Mfotie Njoya E., Munvera A.M., Mkounga P., Nkengfack A.E., McGaw L.J. (2017). Phytochemical analysis with free radical scavenging, nitric oxide inhibition and antiproliferative activity of Sarcocephalus pobeguinii extracts. BMC Compl. Alternative Med..

[20] Oyaizu M., dietetics (1986).

[21] Mamza U., Sodipo O., Abdulrahman F., Khan I. (2015). Evaluation of phytochemical, antimicrobial and toxicity studies of ethanolic stem extract of Phyllanthus amarus Thonn and Schum (Euphorbiacea). The International Journal of Science and Technoledge.

[22] Rajasekaran A., Arivukkarasu R., Murugesh S. (2010). Evaluation of antipyretic activity of ethyl acetate extract of Adenema hyssopifolium G. Don in a rat model. Asian Pac. J. Tropical Med..

[23] D'Amour F.E., Smith D.L. (1941). A method for determining loss of pain sensation. J. Pharmacol. Exp. Therapeut..

[24] Eddy N.B., Leimbach D. (1953). Synthetic analgesics. II. Dithienylbutenyl-and dithienylbutylamines. J. Pharmacol. Exp. Therapeut..

[25] Koster R. (1959).

[26] Khan R.A., Khan M.R., Ahmed M., Sahreen S., Shah N.A., Shah M.S., Bokhari J., Rashid U., Ahmad B., Jan S. (2012). Hepatoprotection with a chloroform extract of Launaea procumbens against CCl4-induced injuries in rats. BMC Compl. Alternative Med..

[27] Toor S.S., Reddy H., Deng S., Hoffmann J., Spangsmark D., Madsen L.B., Holm-Nielsen J.B., Rosendahl L.A. (2013). Hydrothermal liquefaction of Spirulina and Nannochloropsis salina under subcritical and supercritical water conditions. Bioresour. Technol..

[28] Reitman S., Frankel S. (1957). A colorimetric method for the determination of serum glutamic oxalacetic and glutamic pyruvic transaminases. Am. J. Clin. Pathol..

[29] Otto A., Oliver H., Jane M. (1946). A method for the rapid determination of alkaline phosphatase with five cubic millimeters of serum. J. Biol. Chem..

[30] Whitfield J. (2001). Gamma glutamyl transferase. Crit. Rev. Clin. Lab Sci..

[31] Doumas B.T., Kwok-Cheung P.P., Perry B.W., Jendrzejczak B., McComb R.B., Schaffer R., Hause L.L. (1985). Candidate reference method for determination of total bilirubin in serum: development and validation. Clin. Chem..

[32] Bartels H., Böhmer M., Heierli C. (1972). Serum creatinine determination without protein precipitation. Clinica chimica acta; international journal of clinical chemistry.

[33] Fawcett J. (1960). Scott Je. A rapid and precise method for the determination of urea. J. Clin. Pathol..

[34] Chin T.Y., Cacini W., Zmuda M., Quebbemann A. (1980).

[35] Ohkawa H., Ohishi N., Yagi K. (1979). Assay for lipid peroxides in animal tissues by thiobarbituric acid reaction. Anal. Biochem..

[36] Lowery G.H. (1951).

[37] Sedlak J., Lindsay R.H. (1968). Estimation of total, protein-bound, and nonprotein sulfhydryl groups in tissue with Ellman's reagent. Anal. Biochem..

[38] Stephane F.F.Y., Jules B.K.J. (2020). Essential Oils-Bioactive Compounds, New Perspectives and Applications.

[39] Cox-Georgian D., Ramadoss N., Dona C., Basu C. (2019). Therapeutic and medicinal uses of terpenes. Med. Plants: from farm to pharmacy.

[40] Uritu C.M., Mihai C.T., Stanciu G.-D., Dodi G., Alexa-Stratulat T., Luca A., Leon-Constantin M.-M., Stefanescu R., Bild V., Melnic S. (2018).

[41] Baker A.K. (2020).

[42] Park S.-H., Sim Y.-B., Lim S.-S., Kim J.-K., Lee J.-K., Suh H.-W. (2010). Antinociception effect and mechanisms of Campanula punctata extract in the mouse. KOREAN J. PHYSIOL. PHARMACOL..

[43] Chapman C.R., Casey K., Dubner R., Foley K., Gracely R., Reading A. (1985). Pain measurement: an overview. Pain.

[44] Sales A., Felipe L.d.O., Bicas J.L. (2020). Production, properties, and applications of α-terpineol. Food Bioprocess Technol..

[45] Guimarães A.G., Quintans J.S., Quintans‐Júnior L.J. (2013). Monoterpenes with analgesic activity—a systematic review. Phytother Res..

[46] Bouchama A., Abuyassin B., Lehe C., Laitano O., Jay O., O'Connor F.G., Leon L.R. (2022). Classic and exertional heatstroke. Nat. Rev. Dis. Prim..

[47] Zhao X.-X., Lin F.-J., Li H., Li H.-B., Wu D.-T., Geng F., Ma W., Wang Y., Miao B.-H., Gan R.-Y. (2021). Recent advances in bioactive compounds, health functions, and safety concerns of onion (Allium cepa L.). Front. Nutr..

[48] Unsal V., Cicek M., Sabancilar İ. (2021). Toxicity of carbon tetrachloride, free radicals and role of antioxidants. Rev. Environ. Health.

[49] Fahmy M.A., Diab K.A., Abdel-Samie N.S., Omara E.A., Hassan Z.M. (2018). Carbon tetrachloride induced hepato/renal toxicity in experimental mice: antioxidant potential of Egyptian Salvia officinalis L essential oil. Environ. Sci. Pollut. Control Ser..

[50] Aslan A., Gok O., Beyaz S., Ağca C.A., Erman O., Zerek A. (2020). Ellagic acid prevents kidney injury and oxidative damage via regulation of Nrf-2/NF-κB signaling in carbon tetrachloride induced rats. Mol. Biol. Rep..

[51] Ullah H., Khan A., Baig M.W., Ullah N., Ahmed N., Tipu M.K., Ali H., Khan S. (2020). Poncirin attenuates CCL4-induced liver injury through inhibition of oxidative stress and inflammatory cytokines in mice. BMC complementary medicine and therapies.

[52] Ma J.-Q., Ding J., Zhang L., Liu C.-M. (2014). Ursolic acid protects mouse liver against CCl4-induced oxidative stress and inflammation by the MAPK/NF-κB pathway. Environ. Toxicol. Pharmacol..

[53] Ayala A., Muñoz M.F., Argüelles S. (2014).

[54] Pizzimenti S., Ciamporcero E., Daga M., Pettazzoni P., Arcaro A., Cetrangolo G., Minelli R., Dianzani C., Lepore A., Gentile F. (2013). Interaction of aldehydes derived from lipid peroxidation and membrane proteins. Front. Physiol..

[55] Seitz H.K., Bataller R., Cortez-Pinto H., Gao B., Gual A., Lackner C., Mathurin P., Mueller S., Szabo G., Tsukamoto H. (2018). Alcoholic liver disease. Nat. Rev. Dis. Prim..

[56] Adeyemi O.S., Fambegbe M., Daniyan O.R., Nwajei I. (2012). Yoyo Bitters, a polyherbal formulation influenced some biochemical parameters in Wistar rats. J. Basic Clin. Physiol. Pharmacol..

[57] Luft F.C. (2021). Biomarkers and predicting acute kidney injury. Acta Physiol..

[58] Andrade R.J., Chalasani N., Björnsson E.S., Suzuki A., Kullak-Ublick G.A., Watkins P.B., Devarbhavi H., Merz M., Lucena M.I., Kaplowitz N. (2019). Drug-induced liver injury. Nat. Rev. Dis. Prim..

[59] Zafar R., Ali S.M. (1998). Anti-hepatotoxic effects of root and root callus extracts of Cichorium intybus L. J. Ethnopharmacol..

[60] Sadeghi H., Jahanbazi F., Sadeghi H., Omidifar N., Alipoor B., Kokhdan E.P., Mousavipoor S.M., Mousavi-Fard S.H., Doustimotlagh A.H. (2019). Metformin attenuates oxidative stress and liver damage after bile duct ligation in rats. Research in pharmaceutical sciences.

[61] Friedman L., Martin P., Munoz S. (1996). Liver function tests and the objective evaluation of the patient with liver disease. Hepatology: a textbook of liver disease.

[62] Kantola T., Kivistö K.T., Neuvonen P.J. (1998). Grapefruit juice greatly increases serum concentrations of lovastatin and lovastatin acid. Clin. Pharmacol. Therapeut..

[63] Liu K.J., Shi X. (2001). Molecular Mechanisms of Metal Toxicity and Carcinogenesis.

[64] Klop B., do Rego A.T., Cabezas M.C. (2013). Alcohol and plasma triglycerides. Curr. Opin. Lipidol..

[65] Wołyniec W., Kasprowicz K., Giebułtowicz J., Korytowska N., Zorena K., Bartoszewicz M., Rita-Tkachenko P., Renke M., Ratkowski W. (2019). Changes in water soluble uremic toxins and urinary acute kidney injury biomarkers after 10-and 100-km runs. Int. J. Environ. Res. Publ. Health.

[66] Bouzenna H., Hfaiedh N., Giroux-Metges M.-A., Elfeki A., Talarmin H. (2017). Potential protective effects of alpha-pinene against cytotoxicity caused by aspirin in the IEC-6 cells. Biomed. Pharmacother..

[67] Lněničková K., Svobodová H., Skálová L., Ambrož M., Novák F., Matoušková P. (2018). The impact of sesquiterpenes β-caryophyllene oxide and trans-nerolidol on xenobiotic-metabolizing enzymes in mice in vivo. Xenobiotica.

[68] Fraga B.M. (2013). Natural sesquiterpenoids. Nat. Prod. Rep..

[69] Harb A.A., Bustanji Y.K., Abdalla S.S. (2018). Hypocholesterolemic effect of β-caryophyllene in rats fed cholesterol and fat enriched diet. J. Clin. Biochem. Nutr..

[70] Kelany M.E., Abdallah M.A. (2016). Protective effects of combined β-caryophyllene and silymarin against ketoprofen-induced hepatotoxicity in rats. Can. J. Physiol. Pharmacol..

[71] Refaat B., El-Boshy M. (2022). Protective antioxidative and anti-inflammatory actions of β-caryophyllene against sulfasalazine-induced nephrotoxicity in rat. Exp. Biol. Med..

[72] Rahmani H., Moloudi M.R., Hashemi P., Hassanzadeh K., Izadpanah E. (2023). Alpha-Pinene alleviates motor activity in animal model of Huntington's disease via enhancing antioxidant capacity. Neurochem. Res..

